# Surveillance of Side Effects after Two Doses of COVID-19 Vaccines among Patients with Comorbid Conditions: A Sub-Cohort Analysis from Saudi Arabia

**DOI:** 10.3390/medicina58121799

**Published:** 2022-12-06

**Authors:** Tauqeer Hussain Mallhi, Yusra Habib Khan, Muhammad Hammad Butt, Muhammad Salman, Nida Tanveer, Nasser Hadal Alotaibi, Abdulaziz Ibrahim Alzarea, Abdullah Salah Alanazi

**Affiliations:** 1 Department of Clinical Pharmacy, College of Pharmacy, Jouf University, Sakaka 72388, Saudi Arabia; 2 Health Sciences Research Unit, Jouf University, Sakaka 72388, Saudi Arabia; 3 Department of Medicinal Chemistry, Faculty of Pharmacy, Uppsala University, 75123 Uppsala, Sweden; 4 Institute of Pharmacy, Faculty of Pharmaceutical and Allied Health Sciences, Lahore College for Women University, Lahore 54000, Pakistan; 5 Institute of Molecular Cardiology, University of Louisville, Louisville, KY 40202, USA

**Keywords:** COVID-19, side effects, safety, vaccine hesitancy, comorbidities, hypertension, diabetes mellitus, hyperlipidemia, pharmacovigilance

## Abstract

*Background*: Individuals with underlying chronic illnesses have demonstrated considerable hesitancy towards COVID-19 vaccines. These concerns are primarily attributed to their concerns over the safety profile. Real-world data on the safety profile among COVID-19 vaccinees with comorbid conditions are scarce. This study aimed to ascertain the side-effects profile after two doses of COVID-19 vaccines among chronic-disease patients. *Methodology*: A cross-sectional questionnaire-based study was conducted among faculty members with comorbid conditions at a public educational institute in Saudi Arabia. A 20-item questionnaire recorded the demographics and side effects after the two doses of COVID-19 vaccines. The frequency of side effects was recorded following each dose of vaccine, and the association of the side-effects score with the demographics was ascertained through appropriate statistics. *Results*: A total of 204 patients with at least one comorbid condition were included in this study. A total of 24 side effects were reported after the first dose and 22 after second dose of the COVID-19 vaccine. The incidence of at least one side effect was 88.7% and 95.1% after the first and second doses of the vaccine, respectively. The frequent side effects after the first dose were pain at the injection site (63.2%), fatigue (58.8%), fever (47.5%), muscle and joint pain (38.7%), and headache (36.3%). However, pain at the injection site (71.1%), muscle and joint pain (62.7%), headache (49.5%), fever (45.6%), and stress (33.3%) were frequent after the second dose. The average side-effects score was 4.41 ± 4.18 (median: 3, IQR: 1, 6) and 4.79 ± 3.54 (median 4, IQR: 2, 6) after the first and second dose, respectively. Female gender, diabetes mellitus, hypertension, hyperlipidemia, comorbidity > 2, family history of COVID-19, and the AstraZeneca vaccine were significantly associated with higher side-effect scores. Only 35.8% of study participants were satisfied with the safety of COVID-19 vaccines. *Conclusions*: Our analysis showed a high proportion of transient and short-lived side effects of Pfizer and AstraZeneca vaccines among individuals with chronic illnesses. However, the side-effects profile was comparable with the safety reports of phase 3 clinical trials of these vaccines. The frequency of side effects was found to be associated with certain demographics, necessitating the need for further investigations to establish a causal relationship. The current study’s findings will help instill confidence in the COVID-19 vaccines among people living with chronic conditions, overcome vaccine hesitancy, and increase vaccine coverage in this population.

## 1. Introduction

The first case of coronavirus disease (COVID-19) was identified in December 2019 in Wuhan, China. The mortality rate of COVID-19 is lower than that of other coronaviruses such as severe acute respiratory syndrome (SARS) and Middle East respiratory syndrome (MERS). However, its causative pathogen, “severe acute respiratory syndrome coronavirus 2 (SARS-CoV-2)” is considered more contagious than other coronaviruses causing SARS and MERS [1]. Initially, patients with COVID-19 presented with typical manifestations such as fever, fatigue, and respiratory symptoms. However, the rapid distribution of COVID-19 across the globe resulted in several atypical intricacies in various organ systems during the disease course of COVID-19 [2]. Immediate prevention and control measures were taken to curtail the epidemic across the globe. The phenomena of social distancing [3], drug repurposing for the management of patients [4], and the development of vaccines proved to be effective maneuvers to combat the growing encumbrance of COVID-19.

The COVID-19 vaccination is one of the effective preventive measures to curb the encumbrance of the ongoing pandemic [5]. Various health authorities around the globe have endorsed herd immunity as a potential tool against the COVID-19 outbreak. Vaccination against COVID-19 is an essential driver of herd immunity. Despite aggressive and continuous maneuvers to increase vaccine coverage, vaccine hesitancy is still a prominent phenomenon that may impact the process of mass immunization against COVID-19. However, the fear of side effects of the COVID-19 vaccines is considered a dominant factor in vaccine hesitancy [6,7]. The safety of COVID-19 vaccines is of great public health concern and is crucial to neutralize vaccine hesitancy. There has been widespread speculations of serious post-vaccination adverse events. In addition, the safety of these vaccines became a priority concern immediately after a few reports of blood clotting [8], Bell‘s palsy [9], and myocarditis [10] following the administration of specific vaccines.

Healthcare researchers around the globe responded swiftly to the safety profile of the COVID-19 vaccines and conducted various post-marketing real-world surveillance studies of side effects among vaccinees [11,12,13,14]. However, there is a dearth of investigations into post-vaccination side-effects of COVID-19 vaccines among recipients with underlying comorbid conditions. In addition, there are also increased safety concerns regarding the vaccination of people living with underlying chronic conditions [15]. The use of more than one medication and the co-occurrence of multiple health conditions among chronic-disease patients may increase the frequency and severity of the post-vaccination side effects [15,16]. It is pertinent to mention that most of the clinical trials on vaccines have included only stable chronic-disease patients. Moreover, this population accounted for only 15% to 42% of total participants in these trials [17]. Most chronic-disease patients remain reluctant to adopt any therapeutic intervention except the medications they are already receiving. Yi et al. reported that most of the patients with rheumatic diseases were reluctant to receive COVID-19 vaccines due to the fear of side effects and disease flare [18]. These findings are alarming, as patients with rheumatic diseases are at high risk of COVID-19 infection attributed to their compromised immune systems [19]. However, recent investigations have demonstrated satisfactory immunogenicity and an acceptable safety profile of the COVID-19 vaccines among patients with autoimmune inflammatory rheumatic diseases (AIIRDs) [20,21]. The findings of Li et al. also encourage COVID-19 vaccination among rheumatic-disease patients. The authors have reported a satisfactory safety profile of COVID-19 vaccines among individuals with various rheumatic diseases, and indicated that immunosuppressive therapy was not associated with the frequency of side effects [21]. Educating these patients regarding the benefits and safety of COVID-19 vaccines will ensure optimal coverage. Similar results have also been observed in a study conducted on the Saudi population, where adults with chronic conditions demonstrated a low willingness to receive the COVID-19 vaccination [22]. It is expected that people with chronic health conditions will hesitate to receive COVID-19 vaccines, most probably due to fear of side effects. In this context, there is an impetus to inform this population regarding safety profile of the COVID-19 vaccines. Cautious pharmacovigilance is indeed warranted for people with one or more chronic illnesses. This study aimed to ascertain the prevalence of side effects after the first and second doses of COVID-19 vaccines among chronic-disease patients. Moreover, this study determined the relationship between the frequency of side effects and the demographics. The findings may serve to inform the general community regarding the safety of COVID-19 vaccines among individuals with underlying medical conditions.

## 2. Materials and Methods

### 2.1. Ethics Statement

The study protocol was approved by the Local Committee of Bioethics (LCBE) at Jouf University, KSA (Reference: 05-05-43). Informed consent was obtained from all the participants and data were anonymized before analysis.

### 2.2. Study Design, Population, and Location

This study is a sub-cohort analysis of an ongoing project on the pharmacovigilance of the COVID-19 vaccines. This project aimed to collect data on the side effects of COVID-19 vaccines from university students, faculty members, and the general population. Since the side-effects profile of COVID-19 vaccines among chronic-disease patients is lacking in the literature, a sub-group analysis on vaccinees with chronic disease was performed. Considering the dire need for a rapid report on the safety profile of the COVID-19 vaccines among chronic-disease patients, data on faculty members of Jouf University, Saudi Arabia were readily available for analysis. The participants who had received at least two doses of the COVID-19 vaccines, had at least one chronic condition, and consented to participate were included in this study, or otherwise excluded.

### 2.3. Validation and Reliability of Study Instrument

A 20-item questionnaire comprising three sections was constructed by a team of physicians and hospital/community pharmacists. Following the face and content validity, the study instrument was administered to a small sample of 30 participants. The internal consistency of the study tool was estimated through Cronbach‘s alpha at 0.821, indicating the suitability and reliability of the study instrument.

### 2.4. Components of Study Instrument

The study instrument was specifically designed to evaluate the safety profile of COVID-19 vaccines. The questionnaire was initially designed in English. The English version was translated into Arabic with the help of native Arabic speakers using forward–backward translation. The study questionnaire consisted of four sections. Section 1 collected information on demographics including age, gender, marital status, nationality, and education. Section 2 inquired about the chronic conditions present among the study participants. Section 3 evaluated the safety profile of vaccines such as the type of side effect after each dose of vaccine, the onset and duration of side effects, and their management. Section 4 had two questions inquiring about the satisfaction and recommendation of the COVID-19 vaccines among study participants. The list of side effects included all common events reported in clinical trials of vaccines as well as in the previous literature. Moreover, participants were also encouraged to document any other side effect that was not present in the list. All the participants were asked to respond against each side effect on a scale of “Yes” and “No”. The option “Yes” was scored “1”, otherwise “zero”. A cumulative side-effect score of each participant was estimated by adding the total number of side effects. The mean side-effects scores were also estimated after the first and second doses of COVID-19 vaccines. The total number of side effects after the first and second dose was estimated. This resulted in a cumulative side-effect score (CSES) (the presence of each side effect was scored 1). The average side-effect score (ASES) was also estimated after each dose (ASES = (CSES-Dose 1 + CSES-Dose 2)/2). The reported side effects were further stratified into four categories i.e., common (side effects indicated by >50% of the study population), moderately common (30–50%), uncommon (10% to <30%), and rare (<10%). The respondents were also asked to rate the severity of side effects from mild to moderate or severe.

### 2.5. Data Collection

Using a convenient sampling technique, the questionnaire was distributed among the staff of the Jouf University via official emails with regular reminders at predefined intervals. A brief overview of the study was given to the participants. Subsequently, the participants were asked about their volunteer participation in this study and an online consent was obtained with the statement “I agree to participate in this study”. If the participants did not agree, the form was submitted without recording their responses. All questionnaires were checked for completeness and transferred to a Microsoft spreadsheet for cleaning purposes. Initially, all the data were collected but only responses meeting the inclusion criteria were included in the analysis.

### 2.6. Statistical Analysis

All the data were subjected to analysis by Statistical Package for Social Sciences (IBM-SPSS version 25). The data were descriptively presented as the frequency with proportion and mean with standard deviation. The categorical data were compared with the Chi-square test i.e., the comparison of side effects across demographics and types of the COVID-19 vaccine. The independent Student’s t-test and one-way ANOVA compared the mean score between two or more than two groups, respectively. Pearson correlation was used to estimate the relationship between the number of side effects and comorbidities. The coefficient of determination was also estimated through linear regression. A significance level of *p* < 0.05 value was adjusted in all analyses.

## 3. Results

### 3.1. Demographics

A total of 204 patients with at least one comorbid condition were included in the analysis. The age of the participants ranged from 30 to 49 years. The most common comorbid condition was diabetes mellitus (41.2%) followed by hypertension (31.9%) and hyperlipidemia (30.4%). Almost a quarter of the participants (23.5%) had a history of COVID-19 infection. Most of the participants (62.3%) had received two doses of Pfizer vaccine (BioNTech, BNT162b2), and only 17.2% had received two doses of Astra-Zeneca (Oxford, AZD1222). A homologous vaccine regime was used in 79.9% of participants, while 20.1% of participants received a heterologous regime. The post-vaccination COVID-19 infection rate was only 2% in this study (Table 1).

### 3.2. Incidence of Side Effects after COVID-19 Vaccines

The incidence of at least one side effect was 88.7% and 95.1% after the first and second doses of the vaccine, respectively. Pain at the injection site and fatigue were common side effects after the first dose, while pain at the injection site and muscle or joint pain were common side effects after the second dose. The frequent side effects after the first dose were pain at the injection site (63.2%), fatigue (58.8%), fever (47.5%), muscle and joint pain (38.7%), and headache (36.3%). However, pain at the injection site (71.1%), muscle and joint pain (62.7%), headache (49.5%), fever (45.6%), and stress (33.3%) were frequent after the second dose. Most of these side effects occurred within the 6 h following the first dose (45.1%) and second dose (28.4%). Most of the side effects (61.3%) lasted for 48 h among the study participants (Table 2).

### 3.3. Side-Effect Score and its Association with Demographics

A total of 24 side effects were reported after the 1st dose, and 22 after the 2nd dose of the COVID-19 vaccines. The average -effects score was 4.41 ± 4.18 (median: 3, IQR: 1, 6) and 4.79 ± 3.54 (median 4, IQR: 2, 6) after the first and second dose, respectively. However, the cumulative side-effect score after both doses was 4.60 ± 2.93 (median: 4.2, IQR: 2.5, 6.5). The number of comorbidities was found to be positively correlated with side-effects score after the first dose (r = 0.345, R2 = 0.125, *p* < 0.001), second dose (r = 0.230, R2 = 0.053, *p* < 0.001), and both doses (r = 0.390, R2 = 0.152, *p* < 0.001). Female gender, diabetes mellitus, hypertension, hyperlipidemia, comorbidity count more than 2, family history of COVID-19 infection, and the Astra-Zeneca vaccine were significantly associated with higher side-effect scores following the first dose. On the other hand, participants aged 30–49 years, >2 comorbidities, family history of COVID-19 infection, and those who received Pfizer had significantly higher side-effect scores after the second dose (Table 3). It is pertinent to mention that homologous and heterologous vaccine regimes were not associated with the SES (score: 4.83 versus 4.61, *p* = 0.717) (Table 3). Table 4 and Table 5 show that the occurrence of certain side effects (common and moderately common) was statistically associated with various demographic features.

### 3.4. Self-Reported Side Effects Severity and Medication Recommendations

Around 7% of participants reported that the side effects were severe after both doses, while 4.4% and 6.9% indicated the severe nature of side effects after the first and second doses, respectively. Paracetamol was recommended to 44.1% of study participants. However, vitamin C along with paracetamol was recommended in 5.4% of vaccine recipients. Medical consultation was sought by 5.4% of vaccinees due to severe side effects, and such consultation was more frequent after the first dose (3.4%). Only 2% of participants reported healthcare consultation after both doses.

### 3.5. Perception of Respondents towards Safety of Vaccination

Only 35.8% of vaccinated individuals considered the COVID-19 vaccines safe, while 9.3% suggested that they are not safe and 54.9% were not sure about their safety. However, three-fourths (75.5%) of the study population indicated that they recommend vaccines to their family members and friends.

## 4. Discussion

To the best of our knowledge, this is the first study to assess the side-effects profile following two doses of COVID-19 vaccines among individuals with comorbid illnesses in the Northern Region of Saudi Arabia. Given the rapid development of COVID-19 vaccines, the safety and efficacy of these vaccines remain an area of concern [23]. Such concerns are even more profound for patients with comorbid conditions due to the high prevalence of polypharmacy and vaccine hesitancy in this population [15,16,17,22]. Our analysis showed that the safety profile of COVID-19 vaccines among chronic-disease patients is comparable to healthy individuals, and these findings are aligned with the existing claims [24,25]. However, the incidence of at least one side effect was higher in individuals with comorbidities when compared to those without any underlying medical conditions. This result is aligned with the findings of Alemayehu et al. where authors reported that the magnitude of COVID-19 vaccine adverse effects was comparatively higher among participants with comorbid conditions [26]. Considering the higher risks of disease progression or death during COVID-19, the benefits of the COVID-19 vaccination outweigh the risks among patients with underline medical conditions.

Various clinical trials on vaccine development have included 20% to 30% of individuals with underlying medical conditions. The trials on Pfizer [27] and Moderna [28] vaccines showed that the protective effects were similar across the study participants with and without comorbidities [25]. However, the trials on Astra-Zeneca [29,30] and Janssen [31] showed better protective effects among participants without any comorbid condition than those with comorbidities, but the difference was statistically insignificant. It is important to note that mortality was not associated with any vaccine among participants with and without comorbid conditions. Based on these results, The Advisory Committee on Immunization Practices (ACIP) in the USA, The Joint Committee on Vaccination and immunization (JCVI) in the UK, and the WHO Strategic Advisory Group of Experts on Immunization (SAGE) recommend the vaccination for individuals with high-risk comorbidities [25].

Most of the participants in our study experienced at least one side effect following both doses of COVID-19 vaccines. These findings are in concordance with the results of a large surveillance report from the United Arab Emirates (UAE), where participants with comorbidities experienced more side effects than those without any comorbid condition [32]. Other studies among Arab population have also demonstrated significant association of chronic diseases with the development [33], as well as frequency and severity, of postvaccination side effects [34]. However, another study of Turkish healthcare workers revealed no association of chronic illness with the emergence and intensity of side effects following the Sinovac vaccine [35]. This inconsistency in results might be attributed to the differences in the study population and type of vaccines. Moreover, the existing evidence suggests a lower frequency of side effects after Sinovac than the Pfizer and Astra-Zeneca vaccines. Elnaem et al. reported that the frequency of side effects among recipients of the Sinovac vaccine was significantly lower than Pfizer-BioNTech and Oxford-Astra-Zeneca recipients [36]. This might be a contributing factor to a high prevalence of side effects, as all the participants in our study received either Pfizer or Astra-Zeneca vaccines. These findings suggest the relationship between the type of vaccine and the frequency of side effects. He et al. reviewed the comparative efficacy and safety of various COVID-19 vaccines and reported a high prevalence of local and systemic side effects after mRNA (Pfizer) and adenovirus-based (Astra-Zeneca) vaccines [37], and these results are aligned with our findings. Interestingly, the frequency of side effects was slightly higher after the second dose and these findings are in agreement with other investigations [38,39,40]. However, these findings are contradicted by the results of some other studies [41,42] where the proportion of side effects was more profound after the first dose. It is pertinent to mention that these contradictions might be attributed to the variation in the study population, the types of vaccines, and the status of chronic illnesses among participants. Taken together, our analysis confirms the high proportion of side effects among Pfizer and Astra-Zeneca recipients with chronic diseases, particularly in those with diabetes mellitus, hypertension, and hyperlipidemia. Moreover, the frequency of side effects was more among patients with >2 comorbid conditions. These results necessitate the need for more investigations on the correlation of type and number of comorbidities with the safety profile of the COVID-19 vaccines.

The distribution of side effects was similar to that reported in other studies [24,43], where the most commonly reported complaints were pain at the injection site, headache, muscle/joint pain, and fever. Most of these symptoms were reported within 6 h following the jab. The recipients of Astra-Zeneca experienced more side effects as compared to Pfizer vaccinees. Likewise, the participants who received two doses of Pfizer had an average of four side effects while recipients of two doses of Astra-Zeneca had an average of six side effects). These findings corroborate the results of other studies [24,43,44] comparing the frequencies of side effects across the types of vaccines. However, it is important to note that a conclusion on the association of the frequency of side effects with the type of vaccine should not be established, as this such association is markedly affected by the recipient‘s demographics. A recent meta-analysis has indicated a higher incidence of side effects following the administration of the Sputnik vaccine [45]. Since these side effects are self-reported by the vaccinees, several covariates must also be considered while interpreting the results. Considering the relationship of demographics with side-effects score, only gender and age were associated with the incidence of side effects after the first and second dose, respectively. Our analysis showed a higher frequency of side effects among females after the first dose as compared to males. These results are in contrast with the findings of Al Bahrani et al. [46] but in agreement with other investigations [24,47]. The higher frequency of side effects among females is well explained by biological mechanisms such as stronger antibody, innate, and adaptive immune responses among females [48]. It is pertinent to mention that the side-effects score was higher among males after the second dose, but the difference was not significant. Similar to other studies [24], our analysis showed that the side effects were more common among the adult population. These results indicate that the pattern of side effects reported among individuals with chronic conditions was similar to that reported among the healthy population.

In this study, 7% of the study population reported that the side effects were severe after both doses, while 6.9% reported more severe side effects after the second dose. Similar results have been reported by Kang et al. [40], where authors indicated the higher severity of adverse events after the second vaccination dose. However, only 5% of the study participants sought medical consultation due to severe side effects. The severity of side effects was self-reported in our study and might be associated with perceived severity among study participants. Nevertheless, most of the study participants used only paracetamol, indicating the mild nature of side effects. These findings necessitate the need for further investigations on the severity of side effects based on clinicians‘ observations among patients with chronic illnesses.

It is worth mentioning that only one-third of the study population reported that the COVID-19 vaccines are safe. Although the recommendation of the vaccines to family and friends was high in our study, the widespread concerns over the safety of COVID-19 vaccines among this population were considerable. The data from various countries have indicated that concerns about side effects are the main reason for hesitancy toward COVID-19 vaccines [49,50]. Established evidence has suggested widespread speculation on the safety profile of COVID-19 vaccines among individuals living with comorbidities and endorsed the prioritized measures to tackle vaccine hesitancy and to improve vaccine uptake in this population [15,51,52]. The side effects reported in our study were self-limiting and were primarily linked with the provocation of the immune system by the vaccines. The public sharing of these findings may enhance the confidence of individuals with comorbidities in the safety profile of the COVID-19 vaccines. This may result in the acceleration of the vaccine-coverage process. The side effects after the second dose of COVID-19 vaccines can interfere with booster dose uptake [53]. In this context, educating the population on the safety profile will be of paramount importance. Given the progression of the disease, odds of mortality, and complications linked with COVID-19 infection among chronic-disease patients, the vaccination of this population against COVID-19 holds an instrumental position in mass-vaccination campaigns. In this context, the confidence of this population in the vaccines is a major factor leveraging the vaccination success.

### Study Limitations and Strengths

The findings of the current study should be interpreted in light of a few shortcomings. Demerits of convenient sampling, and information bias such as reporting bias and recall bias cannot be disregarded in this study. Since the side effects were self-reported, there is a possibility of incorrect blame, as the study population was also using several medications, and some complaints may have been associated with the use of drugs. The patients with diabetes mellitus, hypertension, hyperlipidemia, and asthma represented most of the study population; therefore, the implications of the findings for patients with other comorbid conditions are limited. The long-term impact of these side effects was not determined in this study, necessitating the need for further investigations in this particular population. Moreover, this study does not provide information on the status of comorbidities, as unstable patients may exaggerate the symptoms after vaccination. Since the safety profile of booster doses of COVID-19 vaccines has been investigated [54], our study does not provide any pharmacovigilance data following the booster dose among chronic-disease patients. Considering the limited number of participants in our analysis, replication and verification of our findings by a larger cohort are warranted. Despite these limitations, our findings are strengthened by a detailed analysis of the side-effect profile of the two doses of COVID-19 vaccines among people having diverse chronic conditions. Since various strong evidence suggests the increased risk of COVID-19, breakthrough infection, and waning vaccine effectiveness among people living with various health conditions [55], optimal vaccination coverage is direly needed in this population. In this context, the findings of the current study will help instill confidence in the COVID-19 vaccines and subsequently increase vaccine coverage in this population. Nevertheless, this study confirms the safety of the COVID-19 vaccines, and its findings can be utilized in creating awareness among this vulnerable population with vaccine hesitancy and ambivalence.

## 5. Conclusions

This study indicated a high proportion of transient and short-lived side effects of Pfizer and Astra-Zeneca vaccines among individuals with chronic illnesses. Pain at the injection site was a common side effect after the first and second doses. Fatigue and muscle or joint pain were the second-most-common side effects after the first and second dose, respectively. Most of these side effects occurred after 6 h of vaccine administration and lasted for 48 h. The number of comorbid conditions was found to be positively correlated with the side-effect score after the first and second doses. Only a few participants reported that the side effects were severe in nature. Although three-fourths of the study population indicated the recommendation of vaccines to their family members, only one-quarter of vaccinees endorsed COVID-19 vaccines as safe. The side-effects profile reported in this study was comparable with the safety reports of phase 3 clinical trials of these vaccines. It is important to note that the side effects reported in this study require further validation, verification, and replication through active pharmacovigilance or qualitative studies. A larger study with random sampling would likely detect the relationship of side effects between demographics, type and severity of comorbid illness, and the type of vaccine. The results of this study may help in solving the ongoing challenge of vaccine hesitancy in this vulnerable population that is nurtured by widespread concerns over safety profile.

## Figures and Tables

**Table 1:** Demographics of study participants.

Variables	Frequency	Percentage
**Age**		
18–29 Years	24	11.8
30–49 Years	105	51.5
≥50 Years	75	36.8
**Gender**		
Male	91	44.6
Female	113	55.4
**Marital status**		
Single	41	20.1
Married	158	77.5
Divorced	2	1.0
Widowed	3	1.5
**Comorbidities**		
Diabetes mellitus	84	41.2
Hypertension	65	31.9
Hyperlipidemia	62	30.4
Asthma	56	27.5
Anemia	3	1.5
Hypothyroidism	12	5.9
Hyperthyroidism	6	2.9
Gout	2	1.0
Seasonal allergy	11	5.4
Peptic ulcer	5	2.5
Congestive heart failure	8	3.9
Eczema	2	1.0
**Comorbidities > 2**		
Yes	38	18.6
No	166	81.4
**Allergic to any vaccine**		
Yes	9	4.4
No	195	95.6
**COVID-19 infection before vaccination**		
Yes	48	23.5
No	156	76.5
**COVID-19 infection in family before vaccination**		
Yes	138	67.6
No	66	32.4
**COVID-19 infection in acquaintance before vaccination**		
Yes	161	78.9
No	43	21.1
**COVID-19 vaccination regime**		
Astra-Zeneca (two doses)	36	17.6
Pfizer (two doses)	127	62.3
Pfizer and Astra-Zeneca	41	20.1
**Type of vaccine during 1st dose**		
Pfizer	168	82.4
Astra-Zeneca	36	17.6
**Type of vaccine during 2nd dose**		
Pfizer	127	62.3
Astra-Zeneca	77	37.7
**Combination of vaccine**		
Homologous regime	163	79.9
Heterologous regime	41	20.1
**COVID-19 infection after vaccination**		
Yes	4	2.0
No	200	98.0

**Table 2:** Frequency of side effects after two doses of COVID-19 vaccines.

	First Dose		Second Dose	
	Frequency	Percentage	Frequency	Percentage
Pain at injection site	129	63.2	145	71.1
Swelling at injection site	51	25.0	30	14.7
Redness at injection site	35	17.2	20	9.8
Headache	74	36.3	101	49.5
Muscle and joint pain	79	38.7	128	62.7
Fatigue	120	58.8	64	31.4
Fever	97	47.5	93	45.6
Stress	78	38.2	68	33.3
Malaise (feeling sick)	42	20.6	12	5.9
Chills	41	20.1	47	23.0
Nausea/vomiting	21	10.3	30	14.7
Diarrhea	22	10.8	31	15.2
Cough	18	8.8	42	20.6
Sore throat	15	7.4	21	10.3
Flu-like symptoms	15	7.4	21	10.3
Loss of smell	11	5.4	25	12.3
Loss of taste	10	4.9	33	16.2
Shortness of breath	17	8.3	50	24.5
Menstrual problems	2	1.0	3	1.5
Chest pain	1	0.5	-	-
Palpitations	1	0.5	-	-
Hair loss	3	1.5	3	1.5
Insomnia	10	4.9	8	3.9
Lymph-node swelling	2	1.0	2	1.0
**Onset of side effects**				
Immediately	26	12.7	9	4.4
Within 6 h	92	45.1	58	28.4
Within 6–12 h	15	7.4	37	18.1
Within 12–24 h	21	10.3	25	12.3
After 24 h	19	9.3	21	10.3
Immediately	26	12.7	9	4.4

**Table 3:** Relationship of demographics with side-effects score.

Variables	First Dose			Second Dose		
	Mean	SD	*p* Value	Mean	SD	*p* Values
**Age**			0.070			**0.004**
18–29 Years	3.75	4.235	24	3.63	2.856	
30–49 Years	5.07	4.091	105	5.56	3.648	
≥50 Years	3.71	4.194	75	4.08	3.356	
**Gender**			**<0.001**			0.729
Male	3.24	3.331		4.89	4.416	
Female	5.35	4.555		4.71	2.641	
**Marital status**			0.939			
Single	4.32	4.077		4.51	3.399	
Married	4.47	4.238		4.92	3.601	
Divorced	4.00	5.657		3.50	3.536	
Widowed	3.00	3.464		2.67	1.528	
**Comorbidities**						
*Diabetes mellitus*			**<0.001**			0.249
No	3.16	3.483		4.55	3.793	
Yes	6.20	4.453		5.13	3.123	
*Hypertension*			**0.036**		0.084	
No	139	3.99		4.50	3.431	
Yes	65	5.31		5.42	3.699	
*Hyperlipidemia*			**0.003**			0.064
No	3.75	3.416		4.49	3.459	
Yes	5.94	5.272		5.48	3.638	
*Asthma*			0.813			0.730
No	4.36	3.393		4.74	3.329	
Yes	4.55	5.806		4.93	4.062	
*Anemia*			0.916			0.698
No	4.41	4.192		4.80	3.555	
Yes	4.67	4.041		4.00	2.000	
*Hypothyroidism*			0.726			0.704
No	4.44	4.160		4.77	3.575	
Yes	4.00	4.671		5.17	2.949	
*Hyperthyroidism*			**<0.001**			**<0.001**
No	4.52	4.200		4.63	3.469	
Yes	1.00	0.000		10.00	0.000	
*Gout*			0.658			0.187
No	4.41	4.199		4.82	3.538	
Yes	5.00	1.414		1.50	0.707	
*Seasonal allergy*			0.319			0.955
No	4.34	4.169		4.79	3.619	
Yes	5.64	4.388		4.82	1.537	
*Peptic ulcer*			0.441			0.995
No	4.43	4.216		4.79	3.537	
Yes	3.80	2.588		4.80	3.899	
*Congestive heart failure*			0.080			0.178
No	4.52	4.207		4.81	3.605	
Yes	1.88	2.475		4.38	0.518	
*Eczema*			0.517			0.263
No	4.43	4.194		4.82	3.541	
Yes	2.50	2.121		2.00	1.414	
**Comorbidities > 2**			**0.007**			**<0.001**
Yes	6.63	5.630		6.66	3.052	
No	3.90	3.605		4.36	3.508	
**Allergic to any vaccine**			0.279			0.766
Yes	5.89	5.302		4.44	2.242	
No	4.34	4.126		4.81	3.587	
**COVID-19 infection before vaccination**			0.141			0.117
Yes	3.75	3.219		4.54	3.246	
No	4.62	4.423		5.60	4.286	
**COVID-19 infection in family before vaccination**			**0.012**			**0.009**
Yes	138	3.80		138	5.21	
No	66	5.68		66	3.91	
**COVID-19 infection in acquaintance before vaccination**			**0.046**			**0.003**
Yes	161	3.96		161	4.76	
No	43	6.09		43	4.91	
**COVID-19 vaccination regime**			**0.005**			0.593
Astra-Zeneca (two doses)	6.44	3.308		5.33	3.033	
Pfizer (two doses)	3.96	4.571		4.69	3.802	
Pfizer and Astra-Zeneca	4.02	2.962		4.61	3.089	
**Type of vaccine during 1st dose**			**0.001**			-
Pfizer	3.98	4.227		-	-	
Astra-Zeneca	6.44	3.308		-	-	
**Type of vaccine during 2nd dose**						**0.047**
Pfizer	-	-		3.96	4.571	
Astra-Zeneca	-	-		5.16	3.337	
**Combination of vaccine**						0.717
Homologous regime	-	-		4.83	3.647	
Heterologous regime	-	-		4.61	3.089	

The **bold** values represent statistical significance.

**Table 4:** Distribution of common and moderately common side effects across demographic features after 1st dose of COVID-19 vaccine.

Variable	Sub-variable	Headache		Injection-Site Pain		Fatigue		Bone-Joint Pain		Stress		Fever	
		No	Yes	No	Yes	No	Yes	No	Yes	No	Yes	No	Yes
		%	%	%	%	%	%	%	%	%	%	%	%
**Gender**	Male	52.0	31.0	5.0	40.0	57.0	35.0	50.0	35.0	4.0	42.0	52.0	36.0
	Female	47.0	68.0	4.0	59.0	42.0	64.0	49.0	64.0	5.0	57.0	47.0	63.0
	*p-value*	0.003 *		0.1		0.003 *		0.036 *		0.6		0.020 *	
**Age**	18–29 Years	13.0	9.0	13.0	10.0	15.0	9.0	1.0	11.0	11.0	12.0	1.0	8.0
	30–49 years	5.0	54.0	54.0	49.0	35.0	62.0	4.0	5.0	41.0	67.0	43.0	59.0
	>50 Years	36.0	36.0	3.0	39.0	48.0	28.0	4.0	31.0	47.0	19.0	41.0	3.0
	*p-value*	0.7		0.5		0.001 *		0.4		<0.001 *		0.1	
**Marital status**	Single	22.0	16.0	2.0	17.0	20.0	2.0	19.0	21.0	20.0	19.0	19.0	20.0
	Married	73.0	83.0	74.0	79.0	76.0	78.0	78.0	75.0	7.0	78.0	77.0	77.0
	Divorced	1.0		1.0	0.0	1.0	0.0	0.0	1.0	0.0	1.0	0.0	
	Widowed	2.0			2.0	2.0	0.0	1.0	1.0	1.0	1.0	1.0	
	*p-value*	0.2		0.4		0.8		1.0		1.0		1.0	
**Allergic to vaccine**	No	95.0	95.0	9.0	95.0	96.0	9.0	95.0	96.0	9.0	94.0	97.0	93.0
	Yes	4.0	4.0		4.0	3.0		4.0	3.0		5.0	2.0	6.0
	*P-value*	0.9		0.8		0.6		0.7		0.7		0.2	
**COVID-19-infected**	No	70.0	86.0	81.0	73.0	78.0	7.0	77.0	74.0	75.0	78.0	7.0	82.0
	Yes	29.0	13.0	18.0	26.0	21.0	2.0	22.0	25.0	24.0	21.0	2.0	17.0
	*P-value*	0.011 *		0.2		0.6		0.6		0.6		0.1	
**Family member infected with COVID-19**	No	25.0	44.0	29.0	34.0	3.0	33.0	25.0	4.0	30.0	35.0	29.0	35.0
	Yes	74.0	55.0	70.0	65.0	6.0	66.0	74.0	5.0	69.0	64.0	70.0	64.0
	*P-value*	0.005 *		0.5		0.7		0.010 *		0.4		0.4	
**Friends infected with COVID-19**	No	21.0	20.0	2.0	17.0	20.0	21.0	20.0	21.0	2.0	17.0	1.0	27.0
	Yes	78.0	79.0	7.0	82.0	79.0	78.0	79.0	78.0	7.0	82.0	8.0	72.0
	*p-value*	0.8		0.1		0.8		0.9		0.4		0.024 *	
**COVID-19 infection after vaccinations**	No	97.0	98.0	98.0	97.0	98.0	97.0	10.0	94.0	99.0	96.0	98.0	97.0
	Yes	2.0	1.0	1.0	2.0	1.0	2.0		5.0	0.0	3.0	1.0	2.0
	*p-value*	0.6		0.6		0.5		0.011 *		0.1		0.9	
**Vaccine type**	AZ two doses	11.0	28.0	1.0	18.0	9.0	23.0	9.0	30.0	18.0	16.0		37.0
	Pfizer two doses	67.0	52.0	6.0	61.0	71.0	55.0	70.0	49.0	66.0	55.0	78.0	44.0
	Pfizer and AZ	20.0	18.0	2.0	20.0	1.0	20.0	2.0	20.0	15.0	28.0	21.0	18.0
	*p-value*	0.009 *		0.9		0.025 *		<0.001 *		0.1		<0.001 *	

* Statistical significance.

**Table 5:** Distribution of common and moderately common side effects across demographic features after 2nd dose of COVID-19 vaccine.

Variable	Sub-Variable	Headache		Injection-Site Pain		Fatigue		Bone-Joint Pain		Stress		Fever	
		No	Yes	No	Yes	No	Yes	No	Yes	No	Yes	No	Yes
**Gender**	Male	50.5	38.6	69.5	34.5	46.1	43.8	48.6	35.9	41.9	50	45.9	43
	Female	49.5	61.4	30.5	65.5	53.9	56.3	51.4	64.1	58.1	50	54.1	57
	*p-value*	0.088		<0.001 *		0.092		0.749		0.273		0.674	
**Age**	18-29 Years	14.6	8.9	0	16.6	15.8	9.4	15.7	3.1	12.5	10.3	17.1	5.4
	30–49 Years	51.5	51.5	30.5	60	43.4	56.3	51.4	51.6	49.3	55.9	45.9	58.1
	>50 Years	34	39.6	69.5	23.4	40.8	34.4	32.9	45.3	38.2	33.8	36.9	36.6
	*p-value*	0.402		<0.001 *		0.021 *		0.155		0.666		0.025 *	
**Marital status**	Single	24.3	15.8	0	28.3	23.7	18	22.1	15.6	20.6	19.1	24.3	15.1
	Married	73.8	81.2	98.3	69	72.4	80.5	74.3	84.4	76.5	79.4	72.1	83.9
	Divorced	1	1	0	1.4	1.3	0.8	1.4	0	1.5	0	0.9	1.1
	Widowed	1	2	1.7	1.4	2.6	0.8	2.1	0	1.5	1.5	2.7	0
	*p-value*	0.472		<0.001 *		0.285		0.488		0.777		0.133	
**Allergic to vaccine**	No	93.2	98	98.3	94.5	97.4	94.5	95	96.9	94.9	97.1	96.4	94.6
	Yes	6.8	2	1.7	5.5	2.6	5.5	5	3.1	5.1	2.9	3.6	5.4
	*p-value*	0.094		0.228		0.545		0.34		0.47		0.539	
**COVID-19-infected**	No	74.8	78.2	74.6	77.2	72.4	78.9	81.4	65.6	100	29.4	73	80.6
	Yes	25.2	21.8	25.4	22.8	27.6	21.1	18.6	34.4	0	70.6	27	19.4
	*p-value*	0.56		0.684		0.014 *		0.287		<0.001 *		0.198	
**Family member infected with COVID-19**	No	34	30.7	39	29.7	48.7	22.7	33.6	29.7	46.3	4.4	25.2	40.9
	Yes	66	69.3	61	70.3	51.3	77.3	66.4	70.3	53.7	95.6	74.8	59.1
	*p-value*	0.616		0.197		0.582		<0.001 *		<0.001 *		0.017 *	
**Friends infected with COVID-19**	No	22.3	19.8	22	20.7	25	18.8	16.4	31.3	27.2	8.8	16.2	26.9
	Yes	77.7	80.2	78	79.3	75	81.3	83.6	68.8	72.8	91.2	83.8	73.1
	*p-value*	0.658		0.831		0.016 *		0.29		0.002 *		0.063	
**COVID-19 infection after vaccinations**	No	98.1	98	100	97.2	98.7	97.7	97.9	98.4	99.3	95.6	99.1	96.8
	Yes	1.9	2	0	2.8	1.3	2.3	2.1	1.6	0.7	4.4	0.9	3.2
	*p-value*	0.984		0.198		0.781		0.609		0.074		0.233	
**Vaccine type**	AZ two doses	17.5	17.8	15.3	18.6	15.8	18.8	17.9	17.2	22.8	7.4	0	38.7
	Pfizer two doses	55.3	69.3	55.9	64.8	73.7	55.5	60	67.2	58.1	70.6	75.7	46.2
	Pfizer and AZ	27.2	12.9	28.8	16.6	10.5	25.8	22.1	15.6	19.1	22.1	24.3	15.1
	*p-value*	0.033 *		0.139		0.521		0.016 *		0.024 *		<0.001 *	

* Statistical significance.

## Data Availability

Not applicable.
